# Effects of Parent Involvement in Homework on Students’ Negative Emotions in Chinese Students: Moderating Role of Parent–Child Communication and Mediating Role of Family Responsibility

**DOI:** 10.3390/bs14121139

**Published:** 2024-11-27

**Authors:** Jiayin Li, Xiaomeng Liu, Deqi Zhu, Haozhe Jiang

**Affiliations:** 1 Institute of Higher Education, East China Normal University, Shanghai 200062, China; 15039074902@163.com; 2 Institute of Education Research, Nanjing University, Nanjing 210023, China; 15036156146@163.com; 3 UCL Institute of Education, University College London, London WC1H 0AL, UK; judyqiqi091@gmail.com; 4 College of Education, Zhejiang University, Hangzhou 310058, China

**Keywords:** students’ negative emotion, parent involvement in homework, parent–child communication, family responsibility

## Abstract

Chinese parents’ involvement in children’s homework has become a hot topic, which not only affects students’ learning but also leads to mental health problems. This study aimed to examine how parent involvement in homework affects students’ negative emotions, focusing on the mediating role of family responsibility and the moderating role of parent–child communication in it. The study uses data from the CFPS 2020 database by Peking University, with a sample size of 6906, resulting in 494 valid observations after data cleaning. Data were analyzed using SPSS 26.0 and SPSS Macro PROCESS, which examined the correlation coefficients, mediation effects, and moderated mediation among the variables. The results found that parent involvement in homework had a significant effect on students’ negative emotions. Family responsibility played a partial mediating role between parent involvement in homework and students’ negative emotions. Parent–child communication played a significant moderating role in the relationship between parent involvement in homework and family responsibility on students’ negative emotions. The results are consistent with the Family Systems Theory, and help to reduce the negative emotions of students and promote the physical and mental health of children.

## 1. Introduction

Students’ negative emotions (SNEs), including anxiety, depression, stress, and frustration, are a growing concern in global education. SNEs affect motivation and academic performance [1], and can lead to deeper psychological issues, including mood disorders and behavioral problems, impacting social adaptability and future careers [2,3]. These mental health issues are linked to academic stress, family environment [4], and social challenges, leading to poor academic performance [5], physical health problems [6], and lower life satisfaction [7], with potential severe consequences like self-harm [8] and violence. Therefore, understanding the mechanisms of parental involvement in homework (PIH) is crucial for developing interventions that promote students’ well-being [9]. PIH plays a key role in students’ learning and emotional development [10,11,12], though its impact differs across cultures. In Western countries, PIH aids self-directed learning through resources and environmental support [13,14]. In China, however, PIH often involves active supervision and problem-solving, driven by educational competition and parental concerns [15,16,17]. While PIH can boost motivation, excessive pressure and high expectations may cause anxiety and depression, hinder independence, and lead to conflicts between parents and children [18,19].

Early research on parental involvement in homework (PIH) highlighted its impact on academic performance. Boonk and Brand-Gruwel found that supportive parental behavior was linked to better academic outcomes and self-handicapping strategies [20,21]. Later studies, such as those by Lerner and Levitt, showed that PIH increased motivation and achievement, especially in Asian students [22,23]. Families with higher family responsibility (FR) had children with better academic outcomes [24]. While PIH is known to positively influence academic and behavioral outcomes, including self-efficacy, research on its effects on students’ negative emotions (SNEs) is limited. Most studies focus on the benefits of PIH [25], overlooking the stress and anxiety caused by over-intervention and intense PIH [26]. This paper examines PIH’s effects on SNE in Chinese compulsory education, aiming to optimize parental involvement for students’ emotional well-being. It proposes strategies to reduce SNEs and enhance both academic and emotional development, offering practical recommendations for educational policies to foster positive interactions between families, schools, and society, promoting students’ holistic development and mental health.

### 1.1. Parent Involvement in Homework and Students’ Negative Emotions

Parents play a crucial role in students’ emotions, and their coping strategies significantly impact students’ negative emotions (SNEs) [27]. Studies have shown that increased homework guidance can exacerbate SNEs such as anxiety and depression in students [28]. Excessive parental involvement and control can worsen anxiety, with high-pressure management styles negatively affecting emotional well-being [29,30]. For instance, strict supervision and high expectations from parents can create pressure, leading to anxiety and low mood [31]. Over-involvement can also reduce students’ self-efficacy by limiting their opportunities for self-directed learning. Chronic SNEs can harm social adjustment, affecting self-esteem and social competence [32]. Research on the relationship between parental involvement in homework (PIH) and student psychology indicates that PIH strategies significantly influence SNEs. Therefore, better management of PIH is essential to reduce anxiety and improve students’ psychological health. Based on this, Hypothesis 1 (H1) suggests that higher levels of PIH are positively associated with higher SNEs, aiming to identify the optimal level of parental involvement that mitigates emotional health issues.

### 1.2. The Mediating Role of Family Responsibility

Family responsibility (FR) plays a crucial role in mediating the relationship between parental involvement in homework (PIH) and students’ negative emotions (SNEs). According to Role Identity Theory (RIT), increased parental participation in children’s homework strengthens their role identity within the family, enhancing their sense of responsibility. Research shows that active parental involvement can reduce students’ homework stress and anxiety through moderate supervision and support [33]. Parents with high FR balance expectations and support, avoiding over-involvement, which helps reduce anxiety and improves self-efficacy [34]. Such parents are more attentive to emotional needs and provide a supportive learning environment, stabilizing students’ emotions through emotional support and appropriate autonomy [35]. However, excessive parental responsibility can lead to stress and emotional dysregulation, negatively affecting both parents’ and children’s emotions [36]. Thus, FR indirectly influences students’ emotional well-being and explains the impact of parental management strategies on SNEs. This study proposes Hypothesis 2 (H2): FR mediates the relationship between PIH and SNEs, suggesting that family responsibility bridges the gap between parental involvement and students’ emotional health. Understanding FR helps parents balance involvement in education with sensitivity to their children’s emotional needs [37].

### 1.3. Moderating Role of Parent–Child Communication

Masselam and Stunkard found that effective parent–child communication (PCC) helps parents better understand their children’s needs, improving their knowledge of and commitment to family responsibility (FR) [38]. Open communication enables parents to better address academic challenges and adopt more proactive, adaptive approaches to FR. Positive PCC allows for timely feedback on homework, fostering positive emotions and better support strategies [39]. Parents with higher autonomous motivation can identify homework difficulties through effective communication, enhancing PIH effectiveness and reducing students’ emotional stress (SNEs) [40].

FR significantly affects SNEs. High FR levels are associated with better academic performance and emotional well-being, as responsible parents tend to use supportive strategies that reduce anxiety and stress in children [41,42]. PCC plays a crucial role in regulating SNEs by helping students manage emotions and reducing anxiety and depression, which enhances academic performance [43,44].

Family structure and parents’ educational philosophy also influence PCC’s impact on SNEs. Single-parent families may face greater SNE challenges due to increased time and energy pressures [45,46]. High-quality PCC can improve self-perception, alleviate SNEs, and reflect better parental responsibility [47]. Therefore, improving PCC should be prioritized in educational interventions.

**Figure 1:**
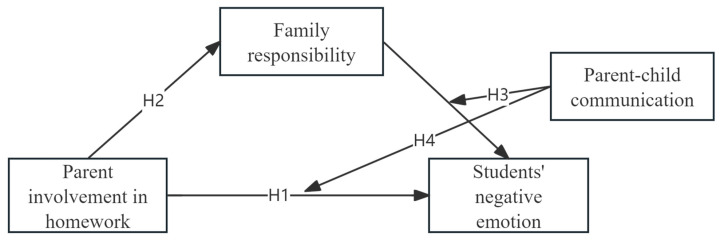
The hypothesized moderated mediation model.

This study proposes Hypothesis 3 (H3): PCC moderates the effect of FR on SNEs, and Hypothesis 4 (H4): PCC moderates the effect of PIH on SNEs, suggesting that better communication enhances parental involvement’s impact on children’s emotional health. The research hypothesis diagram is shown in Figure 1.

Most research has focused on PIH’s impact on academic performance, with less attention on its effects on students’ negative emotions (SNEs). This study addresses this gap by exploring the influence of PIH on SNEs and the mediating role of FR. Additionally, it examines how PCC moderates both PIH’s effect on SNE and the relationship between FR and SNEs. This comprehensive framework highlights the multilevel effects of PCC in homeschooling. The study also helps parents recognize how involvement affects students’ emotions, reducing SNEs, and provides insights for schools and education departments to enhance home–school cooperation, improving parental involvement and family support for students’ overall development and mental health.

## 2. Materials and Methods

### 2.1. Participants

The personal information used in this paper is mainly from the 2020 CFPS database provided by the survey of the Institute of Social Sciences at Peking University, a nationwide, large-scale, multidisciplinary social tracking survey programmed in China. The CFPS was officially launched in 2010 as a nationwide baseline survey, with follow-ups every two years, and the data cover 25 provinces and their administrative divisions, accounting for about 95% of the total mainland population [48]. The fifth survey was conducted between July and December 2020, during which time approximately 62,500 interviews were successfully completed in 31 provinces and districts. The survey provides insights into Chinese family life, including community, household, adult, and child data. A team of 554 interviewers from across the country participated in 176 days of work. As a result of COVID-19, there was a significant shift in data collection methods, with the majority (approximately 89%) of interviews transitioning from face-to-face to telephone interviews. The sample included multiple regions, representing the geographic and economic diversity of China. These regions include economically developed urban areas such as Beijing, Shanghai, and Guangdong, as well as less developed rural areas in provinces such as Gansu and Guizhou.

This study used the Child Parent Proxy (CFPS2020childproxy) from the CFPS2020 public database, which is a newer, higher-quality, and more representative cross-section of data, with an initial sample size of 6906 sample data, but there are fewer valid data in the CFPS data, mainly due to participant attrition, loss of contact, and missing respondent data due to privacy, inaccurate information, or lack of understanding, etc., resulting in missing data. The data were processed by setting inapplicable, missing, and refused responses as missing values, and the remaining valid sample observations were 494 after removing missing values of important variables and outliers. The gender breakdown of the sample was 43.2 percent female and 56.8 percent male, and the household breakdown was 67.8 percent agricultural and 32.2 percent non-agricultural.

### 2.2. Measurement Tools

#### 2.2.1. Parent Involvement in Homework

The PIH was represented by two items, question WF603M, “How often do you ask this child to do homework?” and question WF604M, “How often do you check this child’s homework?” The specific allocations are 1 for “Never”, 2 for “Rarely (once a month)”, 3 for “Occasionally (once a week)”, 4 for “Frequently (2–4 times a week)”, and 5 for “Very often (5–7 times a week)”, and the average is taken as the “PIH” score, which can range from 0 to 5, with higher values indicating greater frequency and recognition. The reliability of this scale was good, with a retest reliability of 0.87. Cronbach’s alpha for this study scale was 0.843 (Table 1).

#### 2.2.2. Parent–Child Communication

The scale consists of two dimensions, question WZ302, “active communication”, and question WF602M, “talking about things at school”. The answers are measured on a 5-point Likert scale (1 = Never, 2 = Seldom, 3 = Occasionally, 4 = Often, 5 = 2–4 times a week, 6 = Seldom, 7 = Rarely, 8 = Rarely, 9 = Often, 10 = Very often) and the average is taken as the “PCC” score. The total score can range from 0 to 5, with higher scores indicating greater frequency and acceptability, and the scale has been validated for use in Chinese populations and has shown strong validity. This scale has previously been validated for use in Chinese populations and has shown strong reliability and validity, and the reliability in this study was relatively high, with a re-test reliability of 0.83. The Cronbach’s alpha for this study’s scale was 0.861 (Table 1).

#### 2.2.3. Family Responsibility

FR was measured by the questions WE104, “Parents are responsible for good or bad grades”; WE106, “Parents are responsible for family harmony”; and WE107, “Parents are responsible for emotional well-being”. Respondents rated each item on a 5-point Likert scale from “Strongly disagree” to “Strongly agree”, and the final average of the three questions was used as the final score for FR. The total score can range from 0 to 5, with higher scores reflecting greater parental FR. This version has previously been validated in a Chinese population and has shown strong reliability and validity, with a re-test reliability of 0.85 and a Cronbach’s alpha for this study’s scale of 0.89 (Table 1).

#### 2.2.4. Students’ Negative Emotions

The Children’s Emotions Scale from the Child Parent Surrogate Questionnaire in CFPS 2020 was used. The scale assesses three key aspects of children’s SNEs: stability, security, and patience. Each dimension is measured by one item, specifically question WF904, “Children get upset immediately when things don’t go their way”; question WF905, “Feel uncomfortable in unfamiliar places, even when adults are around”; and question WF909, “Get upset immediately when not satisfied”.

Responses to each item were rated on a 5-point Likert scale, with 1 being “Never”, 2 being “Occasionally”, 3 being “Sometimes”, 4 being “Often”, and 5 being “Very often“. To calculate the total score of the SNEs, we calculated the overall mean as the final result, which can range from 0 to 5, with higher values indicating higher frequency and endorsement. The reliability of this scale was good, with a retest reliability of 0.87. Cronbach’s alpha for this study scale was 0.858 (Table 1).

To confirm the construct validity, we calculated the composite reliability (CR) for each construct. As Table 1 shows, all CR values were above 0.7. Therefore, our instruments had good construct validity.

To confirm convergent validity, we calculated the average variance extracted (AVE) for each construct. As Table 1 shows, all AVE values were above 0.5. Therefore, our instruments had good convergent validity.

### 2.3. Data Analysis

Data were analyzed using SPSS 26.0 and SPSS macro PROCESS. A Kolmogorov–Smirnov test for normal distribution was used first, followed by a Harman’s one-way analysis of variance to assess common methodological biases. Descriptive statistics were then generated, and Cronbach’s alpha coefficient was used to determine the reliability of the scale. Pearson’s correlation coefficient was then calculated to examine the relationship between the variables. To summaries the analyses, PROCESS (model 15) was used to examine the relationship between perceived SNEs, PIH, FR, and PCC. Statistical significance was defined as a two-tailed *p*-value < 0.05.

## 3. Results

### 3.1. Common Method Bias Tests

In order to ensure the rigor of the study, it was necessary to test the normal distribution of all variables in the predictive and formal tests before data analysis began. Using the Kolmogorov–Smirnov test, it was determined that all continuous variables in the pre-test and normality tests conformed to a normal distribution (all *p*-values were significantly greater than 0.05).

In this study, the Harman one-factor test [49] was used to assess the presence of common methodological biases, following the methodology outlined by Baumgartner and Pieters. The analysis identified four factors, each with an eigenvalue greater than 1. The first factor accounted for 23.753% of the total variance, which is below the commonly used critical threshold of 40%, and it was therefore considered unlikely that the study was affected by common methodological bias, which is within acceptable limits.

### 3.2. Descriptive Statistics and Correlations

Table 2 shows the mean, standard deviation, and correlation coefficients of SNEs, PIH, FR, and PCC. The results show significant correlations between all variables. Specifically, SNEs showed a weak positive correlation with PCC (r = 0.353, *p* < 0.001) and FR (r = 0.239, *p* < 0.001) and a strong positive correlation with PIH (r = 0.797, *p* < 0.001). There was also a positive correlation between FR and PIH (Table 2).

### 3.3. Mediation Analysis

Using PIH as the independent variable and SNE as the dependent variable, this study examined whether FR plays a mediating role between PIH and SNEs, and also examined whether PCC is able to regulate the relationship between them. Therefore, this study examined whether the mediation model with regulation is valid.

In order to test whether the mediated effect model with moderation is valid, this study first conducted a mediated effect test to investigate whether FR mediates between PIH and SNEs. The mediation model analysis and the mediation effect test were conducted using Model 4 of the SPSS macro program Process V3.3 prepared by Hayes, using the nonparametric percentile bootstrap method of bias correction, sampling 5000 times, and selecting the 95% departmental confidence interval, which means that the mediation effect is significant if the standardized path coefficient 95% CI does not include zero.

Table 3 shows that the total effect of PIH on the children’s SNEs was 0.687, the direct effect was 0.612, and the indirect effect was 0.075, with the indirect effect accounting for 9.74% of the total effect. The mediator includes two pathways: pathway 1 (PIH→SNE), with an effect value of 0.612, and pathway 2 (PIH→FR→SNE), with an indirect effect of 0.075. Neither interval mentioned above includes 0, indicating that the mediator effect is significant (Table 3). The direct effect test and the indirect effect test of the mediation effect further verified H1 and H2.

### 3.4. Mediation Analysis with Regulation

The above study shows that FR plays a partial mediating role between PIH and SNEs. Next, PCC was included in the model to explore whether PCC can play a moderating role in it. Model 15 of SPSS macro program process v3.3 was adopted, the non-parametric percentile bootstrap method with bias correction was used to sample 5000 times, and the 95% confidence level was selected for model analysis and moderated effects test.

**Figure 2:**
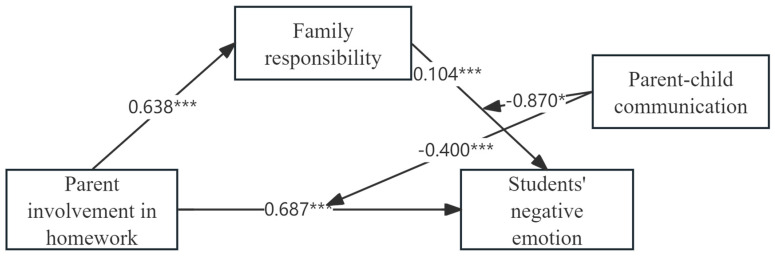
The final moderated mediation model. Notes: * <0.05; *** <0.001.

Table 4 shows that the effects of PIH (β = 0.79, *p* < 0.001), FR (β = 0.104, *p* < 0.001), and PCC (β = 0.136, *p* < 0.001) on SNEs were significant, and the interactions of PIH and PCC (β = −0.4, *p* < 0.001) and of FR and PCC (β = −0.87, *p* < 0.05) on SNE prediction were significant (Table 4). Thus, the mediating role of PIH in the second half of the pathway and the direct effect of having moderation in influencing SNEs via FR was significant (see Figure 2), further validating H4.

**Figure 3:**
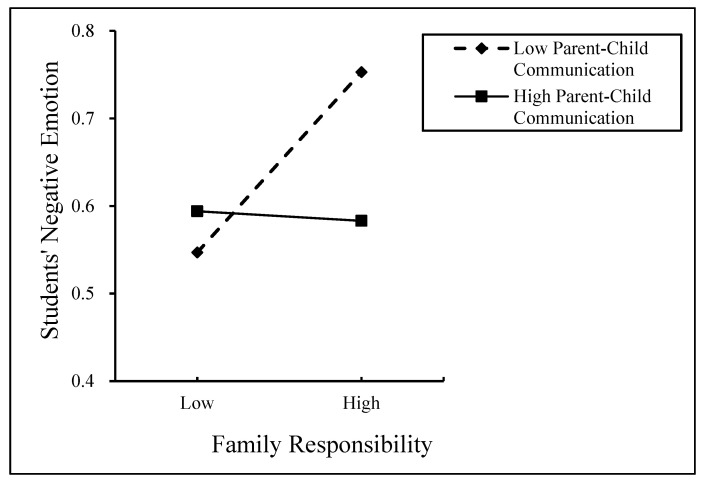
Moderating effect. The moderating role of parent–child communication between family responsibility and students’ negative emotions.

**Figure 4:**
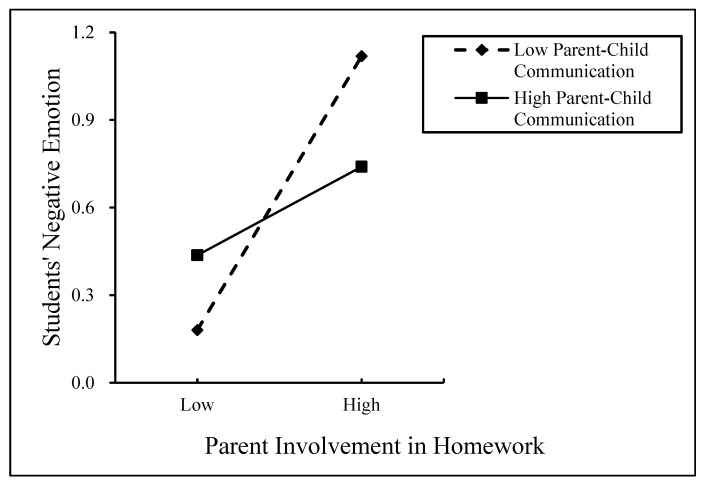
The moderating role of parent–child communication between homework management and students’ negative emotions.

The moderating effect of PCC between FR and SNEs is shown in Figure 3, and the moderating effect of PCC between PIH and SNEs is shown in Figure 4. As can be seen in Figure 3, the predictive effect of FR on the SNEs of students from low-PCC families (β = 0.121, *p* < 0.001) was greater than that of the SNEs of students from high-PCC families, which had no predictive effect. The predictive effect of PIH on SNEs for students from low-PCC families (β = 0.870, *p* < 0.001) was greater than that on SNEs for students from high-PCC families (β = 0.281, *p* < 0.001), suggesting that PIH for low PCC has a more positive effect on SNEs than PIH for high PCC.

## 4. Discussion

### 4.1. Impact of Parental Involvement in Homework on Students’ Negative Emotions

The study found the effect of PIH on SNE: the higher the degree of PIH, the more likely students were to develop negative emotions (in support of H1). This finding is consistent with previous studies that have shown a strong relationship between the level of PIH and students’ emotional state [10,11,12,50], and that PIH can effectively reduce students’ anxiety levels and help them maintain a more positive mood in the face of academic stress [10,11,12]. Excessive PIH is often accompanied by high expectations and demands, and such expectations can, to some extent, be transformed into psychological pressure for students [18,19,51]. According to Self-Determination Theory (SDT), an individual’s intrinsic motivation can be inhibited when he or she feels external pressure, which in turn triggers anxiety and frustration [26]. This psychological state makes it more difficult for students to cope with academic challenges and increases the risk of academic failure [52]. Excessive parental intervention can inhibit students’ autonomy, leading to dependence on parental guidance, weakening independent problem-solving skills, reducing self-confidence, and increasing anxiety. Therefore, effective home education should strike a balance between supporting and encouraging autonomy to help students build self-confidence and increase independence to better cope with academic challenges.

### 4.2. Mediating Role of Family Responsibility

Our study found a mediating role for FR between PIH and SNEs (supporting H2), but the indirect effect was much smaller than the direct effect. First, increased FR not only boosted students’ self-confidence but also promoted self-efficacy [34]. Specifically, when students perceived FR from their parents, they were more likely to view the learning task as their responsibility and to actively participate in the tasks, thus reducing anxiety and frustration and maintaining a positive and optimistic attitude [34,53]. On the other hand, FR is manifested not only in the expectations and support of family members [37] but also in the shaping of the overall family atmosphere. A responsible family environment provides emotional support and active participation in the child’s learning process, and this support helps students maintain positive emotions, which in turn improves motivation and academic performance [24,54]. However, the direct link between FR and pupils’ emotions is weak. Although FR promotes parental involvement and support, this support does not always directly improve students’ emotional state; the effect of FR on mood is more likely to be indirect through its effect on students’ motivation and attitudes towards learning. Students’ emotions are moderated not only by family factors but also by a variety of factors such as emotional regulation skills and peer relationships. Therefore, the manifestations of FR may vary in different families, and there may be individual differences in how different students perceive and respond to FR. Some students may experience positive effects, while others may experience stress, which may influence the effects. Thus, our findings not only confirm the mediating role of FR between parental involvement and students’ emotions but also suggest a more novel mechanism of influence pathways for further research on this relationship. In addition, we highlight the importance of FR in the home education environment, particularly in support of pupils’ emotional and academic development.

### 4.3. Moderating Role of Parent–Child Communication

In addition, the present study found that PCC played an important moderating role in the relationship between PIH and FR on SNEs (supporting H3 and H4).

High-quality PCC may have mitigated the negative effects of PIH on SNEs, which is consistent with the findings of existing studies that families with higher PCC have more emotional support and interaction between parents and children, and that children’s emotions are regulated and channeled in a timely manner [38,55]. When parents engage in homework management, children may be able to better understand and accept this behavior because they feel cared for and supported by their parents. This emotional security may mitigate the negative emotional effects of homework management [40,56]. Therefore, in families with higher parent–child communication, the effect of PIH on SNEs is attenuated and may not even have a significant negative effect. In contrast, in families with low parent–child communication, where there is a lack of effective emotional interaction and communication between parents and children, PIH may manifest more as supervision and control, and because of the lack of sufficient emotional support, the child may perceive homework management as an additional source of stress, leading to a more significant increase in SNEs [30,31].

Similarly, the predictive effect of FR on SNEs was more significant in families with low PCC. The effect of family responsibilities on students’ negative emotions was more significant in families with low PCC, mainly due to the lack of emotional support and effective communication [37]. In such family environments, students may feel isolated and unable to share their stress with their parents when taking on family responsibilities, leading to increased psychological distress [38]. The lack of parental care and guidance makes it difficult for students to regulate their emotions, increasing the risk of negative emotions such as anxiety and depression. In addition, students’ conflict between self-requirement and emotional dependence exacerbated emotional distress. In contrast, good PCC significantly improved parents’ FR, leading them to be more actively involved in their children’s learning and lives [41,42,57], and in turn to care for and support their children’s mental health. Families with strong FR tend to provide more emotional support to students, reducing their negative emotions and psychological problems. However, a lack of responsibility can lead to students feeling lonely and helpless. Quality PCC plays a moderating role in this process and can mitigate the negative effects of responsibility deficit by helping students to express their confusion and receive emotional support. Empirical studies have shown that good parent–child communication is closely related to students’ psychological well-being [58], with FR playing a mediating role. Therefore, promoting quality PCC will not only improve FR but also more effectively support students’ mental health.

The quality of PCC not only affects students’ emotional state but also has a profound impact on their socio-emotional competence [59]. Good communication skills and emotional support can enhance students’ social adjustment and future career potential. For example, positive family interactions can help develop students’ self-confidence and problem-solving skills, leading to better performance in society [46]. Therefore, parents can reduce parent–child SNEs in homework completion through positive communication and emotional support [60], thereby promoting students’ psychological health. China’s compulsory education emphasizes educational cooperation between families and schools, so teachers and parents should jointly pay attention to students’ psychological status, strengthen home–school communication [61], and increase parents’ relevant information about their children, thus improving the quality of homework help and PCC at a later stage.

### 4.4. Theoretical Contributions and Practical Implications

This study contributes theoretically by supporting the mediational model of regulation and highlighting the role of parent–child communication in family dynamics, aligned with Family Systems Theory and Bronfenbrenner’s Ecosystem Theory [62]. It also enriches Social Cognitive Theory by emphasizing FR as a mediating variable. Practically, the study underscores the importance of FR and PCC in students’ mental health and academic success, offering insights for educators and policymakers. Interventions such as parent training and flexible parental involvement mechanisms can enhance home–school collaboration, supporting students’ motivation, mental health, and educational outcomes.

### 4.5. Research Limitations and Perspectives

This study has limitations related to sample selection and data collection. The sample, limited to the Chinese population, affects generalizability, and reliance on self-report data may introduce social desirability bias. Future research should diversify samples and data collection methods, exploring factors like family structure and cultural background to enhance external validity. Longitudinal studies can capture dynamic relationships, while experimental designs can establish causal links. Mixed-methods research will strengthen validity. In conclusion, further empirical studies are needed to explore complex mechanisms and improve parental homework involvement practices.

## 5. Conclusions

In this study, we investigated the effects of PIH on SNEs and revealed the moderating role of PCC and mediating roles of FR. The results showed that PIH had a significant effect on SNEs, and FR played a partial mediating role in this process, while PCC played an important moderating role in the relationship between PIH and FR. In particular, high-quality PCC could attenuate the negative effects of PIH on SNEs. Therefore, improving the quality of PCC can not only reduce the negative effects of PIH on students’ emotional distress but also further alleviate students’ negative emotional experiences by improving FR. In conclusion, good parent–child interactions and effective PIH can reduce students’ negative emotion, thereby reducing childhood mental illness and promoting healthy physical and mental development.

## Figures and Tables

**Table 1:** The reliability and validity testing.

Variable	Items	Item Number	α	Loading	CR	AVE
Students’ negative emotions	Things going wrong quickly makes me unhappy.	WF904	0.858	0.812	0.891	0.555
	Feeling uneasy in unfamiliar places.	WF905		0.568		
	Instantly show displeasure when dissatisfied.	WF909		0.826		
Parent–child communication	Discuss matters related to school.	WF602M	0.861	0.77	0.757	0.609
	Initiate communication.	WZ302		0.791		
Family responsibility	Good or bad grades are the parents’ responsibility.	WE104	0.89	0.619	0.792	0.563
	Family harmony is the parents’ responsibility.	WE106		0.82		
	Emotional happiness is the parents’ responsibility.	WE107		0.796		
Parent involvement in homework	Request to complete the assignment.	WF603M	0.843	0.711	0.722	0.565
	Parents check the homework.	WF604M		0.712		

α: The Cronbach’s alpha coefficient, CR: the composite reliability, AVE: the average variance extracted.

**Table 2:** Means, standard deviations, and correlations.

Variable	M	SD	1	2	3	4
1. Parent involvement in homework	3.535	1.239	1			
2. Parent–child communication	3.468	0.977	0.368 ***	1		
3. Family responsibility	3.369	0.757	0.452 ***	0.115 ***	1	
4. Students’ negative emotions	3.221	0.847	0.797 ***	0.353 ***	0.239 ***	1

Notes. N = 496. *** <0.001; all tests were two-tailed.

**Table 3:** Mediation effect test analysis.

Effect	Path Relationship
Direct effect	Parent involvement in homework→Students’ negative emotions
Indirect effect	Parent involvement in homework→Family responsibility→Students’ negative emotions
Total effect	

Notes. S.E. = standard error; LLCI = lower-level confidence interval; ULCI = upper-level confidence interval.

**Table 4:** Moderated mediation test between different variables.

Variable	Students’ Negative Emotion				
	β	S.E.	t	LLCI	ULCI
Household registration	−0.008	0.051	−0.155	−0.108	0.093
Gender	−0.024	0.048	−0.504	−0.118	0.07
Parent involvement in homework	0.79	0.054	14.689 ***	0.684	0.895
Family responsibility	0.104	0.03	3.412 ***	0.044	0.164
Parent–child communication	0.136	0.04	3.421 ***	0.058	0.214
Parent involvement in homework × Parent–child communication	−0.4	0.045	−8.872 ***	−0.489	−0.312
Family responsibility × Parent–child communication	−0.87	0.038	−2.287 *	−0.162	−0.012
R2	0.425				
F	51.223 ***				
P	0				

Notes: * <0.05; *** <0.001; S.E. = standard error; LLCI = lower-level confidence interval; ULCI = upper-level confidence interval.

## Data Availability

The data in this study are public databases. The data presented in this study are available on request from the corresponding author.
